# Plasma fetal bile acids 7α-hydroxy-3-oxochol-4-en-24-oic acid and 3-oxachola-4,6-dien-24-oic acid indicate severity of liver cirrhosis

**DOI:** 10.1038/s41598-021-87921-5

**Published:** 2021-04-15

**Authors:** Tudor Mocan, Dong Wook Kang, Billy J. Molloy, Hyeonho Jeon, Zeno A. Spârchez, Diren Beyoğlu, Jeffrey R. Idle

**Affiliations:** 1 3rd Medical Clinic, Iuliu Hatieganu University of Medicine and Pharmacy, Cluj-Napoca, Romania; 2 Department of Pharmaceutical Science and Technology, College of Health and Medical Science, Catholic University of Daegu, Gyeongsan-si, Gyeongsangbuk-do 38430 Republic of Korea; 3 Wilmslow, SK9 4AX Waters UK UK; 4 Division of Systems Pharmacology and Pharmacogenomics, Samuel J. and Joan B. Williamson Institute, Arnold and Marie Schwartz College of Pharmacy and Health Sciences, Long Island University, Brooklyn, NY 11201 USA; 5 Department of BioMedical Research, University of Bern, 3008 Bern, Switzerland

**Keywords:** Biomarkers, Gastroenterology

## Abstract

Two 3-oxo-Δ^4^ fetal bile acids, 3-oxachola-4,6-dien-24-oic acid (**1**) and 7α-hydroxy-3-oxochol-4-en-24-oic acid (**2**), occur normally in the human fetus but remain elevated in neonates and children with severe cholestatic liver disease due to an autosomal recessive inborn error of metabolism affecting Δ^4^-3-oxo-steroid 5β-reductase (AKR1D1). Relatively little is known about **1** and **2** in adult patients with liver disease. The chemical synthesis of **1** and **2** is therefore described and their quantitation in plasma by ultrarapid chromatography-triple quadrupole mass spectrometry. Plasma concentrations of **1** and **2** were investigated in 25 adult patients with varying degrees of liver cirrhosis with and without hepatocellular carcinoma (HCC). Highly statistically significant correlations (P < 0.0001) were found between severity of liver cirrhosis, determined by the Child–Pugh and MELD scores, with plasma **1** and **2** concentrations, both alone and combined. The presence of HCC did not influence these correlations. Plasma cholic, chenodeoxycholic, deoxycholic, lithocholic or ursodeoxycholic acids, free and as their glycine or taurine conjugates, did not correlate with Child–Pugh or MELD score when corrected for multiple comparisons. These findings demonstrate that plasma levels of fetal bile acids 3-oxachola-4,6-dien-24-oic acid and 7α-hydroxy-3-oxochol-4-en-24-oic acid and likely deteriorating AKR1D1 activity indicate the severity of liver cirrhosis measured by the Child–Pugh and MELD scores.

## Introduction

Over recent decades, the global prevalence of liver disease has continuously risen until it is now one of the leading causes of morbidity and mortality, with liver fibrosis, liver cirrhosis and progression to hepatocellular carcinoma (HCC) on the rise^1^. In 2017, cirrhosis caused 440,000 deaths in females and 883,000 in males^2^. Despite the use of vaccines and therapeutic agents to reduce hepatitis B and C virus infection that contribute to the burden of liver disease, over-nutrition, obesity, metabolic syndrome and alcohol consumption have become principal causes of liver disease^2, 3^. Hepatic fat overload (steatosis) occurs commonly and can progress to nonalcoholic steatohepatitis (NASH)^4^ and alcoholic steatohepatitis (ASH)^5^, in the case of excessive alcohol consumption. Liver disease advances from stage to stage and therefore procedures have arisen for monitoring this progression with the aim of reducing the incidence of end-stage liver disease and HCC^4^. One such strategy involves the search for biomarkers indicative of disease stage. Mass spectrometry-based metabolomics has proven to be an excellent tool with which to examine premalignant liver disease and its progression to HCC^6^. It had been reported that the two fetal bile acids 3-oxachola-4,6-dien-24-oic acid (**1**) and 7α-hydroxy-3-oxochol-4-en-24-oic acid (**2**), known collectively as 3-oxo-Δ^4^ bile acids, were elevated in plasma^7, 8^ and urine^8^ of HCC patients relative to various control groups, including patients with liver cirrhosis.

The excretion of 3-oxo-Δ^4^ bile acids normally attenuates within the first few days after birth^9^. Elevated 3-oxo-Δ^4^ bile acids in neonates and children with severe cholestatic liver disease was at first interpreted as being due to an autosomal recessive inborn error of metabolism that affected Δ^4^-3-oxo-steroid 5β-reductase^10, 11^ (now known as AKR1D1). The augmentation of fetal bile acids **1** and **2** in urine of infants with hepatobiliary disease was considered to indicate a poor prognosis^12^. It was also proposed that 3-oxo-Δ^4^-steroid-5β-reductase reduced activity could have non-genetic origins, such as altered hepatocyte redox state or unusual sensitivity of the enzyme to liver damage. Therefore, it was anticipated that 3-oxo-Δ^4^ bile acids could be expected to be found in other severe liver diseases^10^. This proposition has implications for the observation of 3-oxo-Δ^4^ bile acids in adult patients with liver disease.

**Figure 1:**
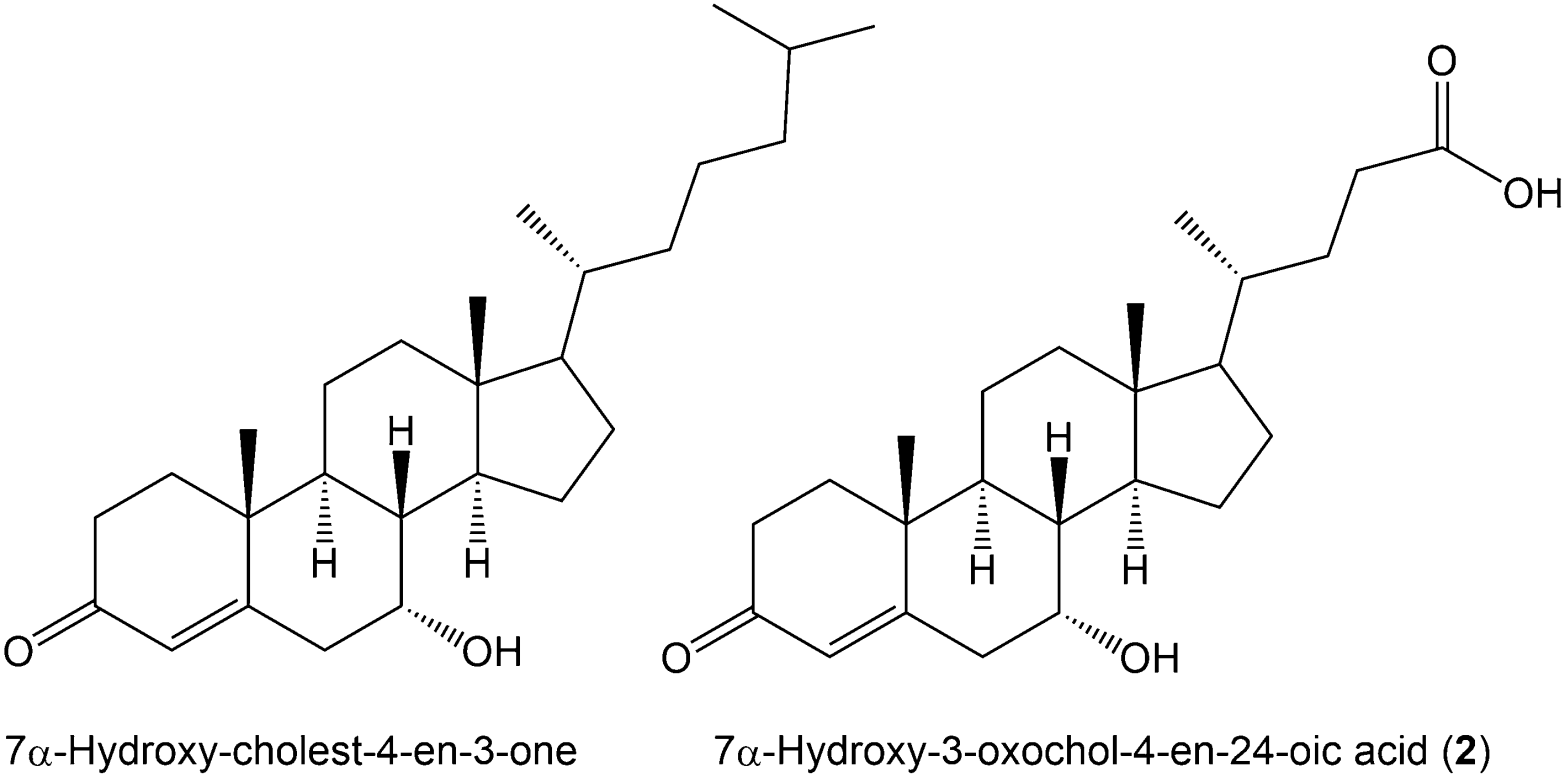
7α-Hydroxy-cholest-4-en-3-one formed from 7α-hydroxycholesterol in the neutral bile acid synthesis pathway and 7α-hydroxy-3-oxochol-4-en-24-oic acid (**2**) formed in the acidic bile acid synthesis pathway.

The synthesis of bile acids from cholesterol proceeds via two alternative pathways, the first of which is the classical “neutral” pathway involving cholesterol 7α-hydroxylation by CYP7A1 with only the final steps entailing creation of the acidic side-chain and leading to cholic acid (CA) and chenodeoxycholic acid (CDCA). In the alternative “acidic” pathway, formation of the acidic side-chain is the first step involving CYP27A1 that finally leads to the synthesis of CDCA^13^. Figure 1 shows 7α-hydroxy-cholest-4-en-3-one that is formed from 7α-hydroxycholesterol in the neutral pathway and the fetal bile acid 7α-hydroxy-3-oxochol-4-en-24-oic acid (**2**) that is formed in the acidic pathway. It is noteworthy that both these species are metabolized by AKR1D1 (Δ^4^-3-oxosteroid 5β-reductase)^11, 13^.

It has been reported that AKR1D1 hepatic expression in adults decreased with progression of steatosis, fibrosis and inflammation and in type 2 diabetic patients^14^. Defective AKR1D1 activity results in elevated 3-oxo-Δ^4^ bile acids in neonates^11^. Whether or not this occurs in adults with liver disease is not known.

We wished to understand if plasma concentrations of 3-oxo-Δ^4^ bile acids could inform regarding the progression of liver cirrhosis to HCC. Because authentic standards of **1** and **2** are not readily available, we describe here their chemical synthesis from chenodeoxycholic acid. Moreover, quantitation of **1** and **2** has hitherto be achieved using gas chromatography-mass spectrometry (GC–MS) methodologies with complex sample preparation and derivatization. We therefore describe a simple and rapid ultraperformance liquid chromatography-tandem mass spectrometry (UPLC-TQMS) assay for these two fetal bile acids in order to promote further research in this area. Importantly, we investigated 15 liver cirrhosis patients with severity measured by Child–Pugh scores^15^ of 5–15 (A-C) together with ten cirrhotic patients with HCC and Child–Pugh scores of 5–11. Using the UPLC-TQMS assay based upon authentic standards for **1** and **2**, the concentration of these fetal bile acids together with 15 conjugated and free primary and secondary bile acids was determined in plasma from these 25 patients. The findings indicate that plasma fetal bile acids **1** and **2**, but not the common bile acids, increase statistically significantly with mounting severity of cirrhosis.

## Results

**Figure 2:**
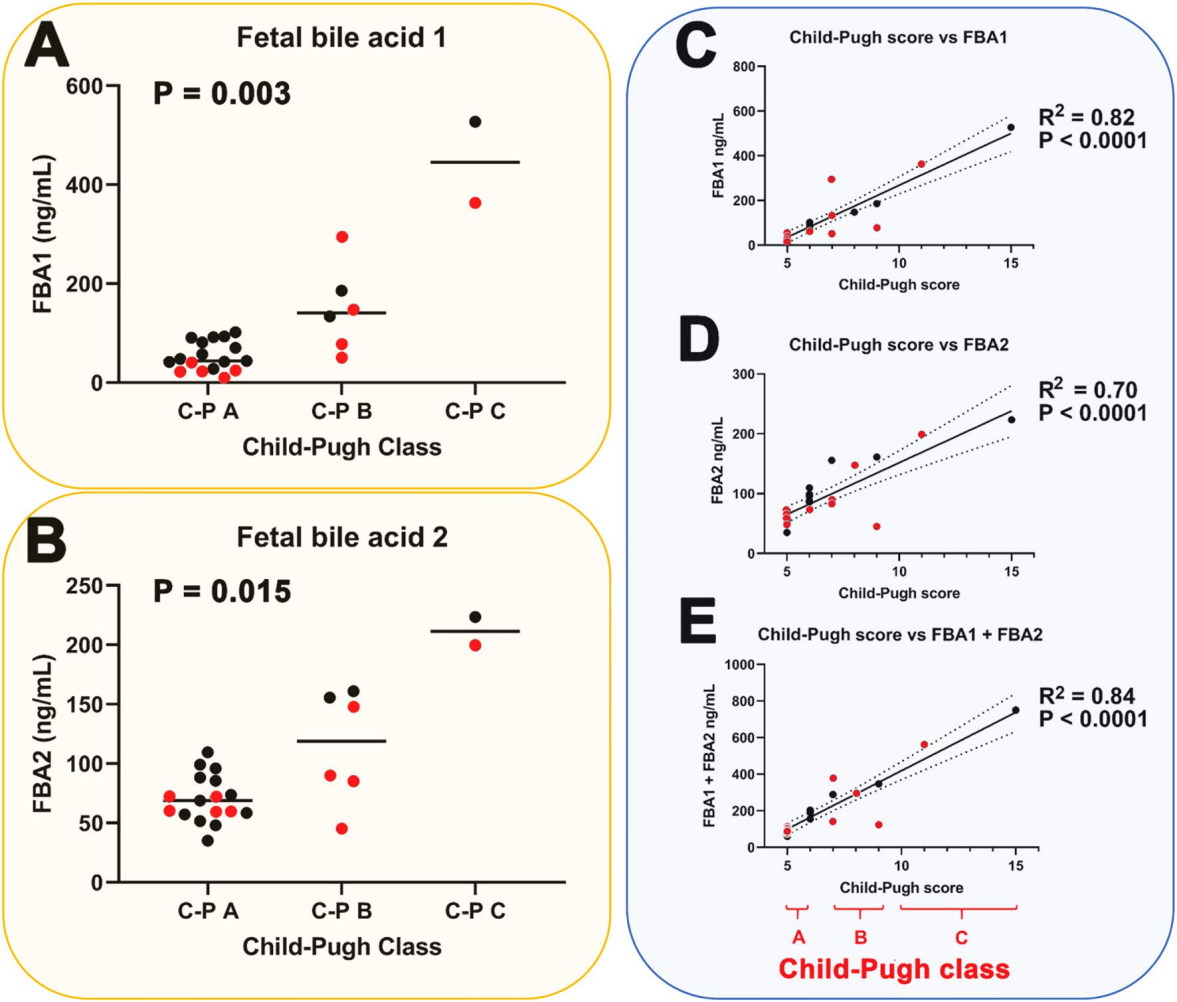
Statistically significant relationship (nonparametric Kruskal–Wallis test) between Child–Pugh class and (**A**) fetal bile acid **1** (FBA1) plasma concentration (ng/mL), (**B**) fetal bile acid **2** (FBA2) plasma concentration (ng/mL) and statistically significant correlations (simple linear regression) between Child–Pugh score and (**C**) FBA1 plasma concentration, (**D**) FBA2 plasma concentration and (**E**) the sum of FBA1 and FBA2 plasma concentrations. C-P mean Child–Pugh. Black symbols represent LC patients without HCC and red symbols represent LC patients with HCC. Dotted lines represent 95% confidence intervals.

Twenty-five patients with LC, comprising 10 with HCC and 15 without, were investigated. The severity of cirrhosis was represented across the full range of Child–Pugh scores^16^ from 5 to 15 in these patients. Clinical details of the ten HCC and 15 LC patients are given in Tables 1 and 2, respectively. Table 3 shows a comparison of clinical data between LC and HCC patients. No confounders were apparent from these data. Of note is the observation that both fetal bile acids **1** and **2** were detected and quantitated in plasma from all 25 cirrhotic patients. In the current investigation, all 25 patients investigated had varying degrees of LC. Examination of their plasma by UPLC-QTOFMS yielded concentrations of fetal bile acids **1** and **2** and we analyzed the findings in relation to both Child–Pugh classes A, B and C together with the Child–Pugh scores from 5 to 15, which represent the severity of the cirrhosis^16^. Figure 2 displays the relationships between Child–Pugh class and score with plasma concentrations of fetal bile acids **1** and **2**. Comparison between Child–Pugh A and Child–Pugh B/C for all cirrhotic patients with and without HCC revealed statistically significant differences for the plasma concentrations of both fetal bile acid **1** (median values 49 and 249, respectively; P < 0.0001) and fetal bile acid **2** (median values 70 and 161, respectively; P = 0.002).

**Table 1:** Hepatocellular carcinoma patient details.

Patient	Sex	Age (years)	Etiology	CT/MRI	Tumor number	Tumor size (mm)	EV	Asc	Enc	BCLC	C-P	AFP	Bili	Alb
1	M	67	HBV/ Eth	HCC/CAC	1	56	No	No	No	C	5	5.5	1.3	3.9
2	M	68	Eth	HCC	3	50, 26, 12	No	Grade I	Yes	B	8	8.0	3.8	3.0
3	M	66	Eth	HCC	7	–	No	Grade I	No	B	7	3.7	2	3.5
4	M	47	HBV/ HDV	HCC	2	32/27, 12	No	No	No	A	5	42	1.1	4.4
5	F	79	HCV	HCC	2	36, 15	Grade II	No	No	A	7	33	2.1	3.4
6	F	73	HCV	HCC	1	45	No	No	No	A	5	> 400	0.8	4.2
7	M	66	HBV	HCC/Mets	2	25, 15	No	No	No	A	5	9.3	1.4	2.5
8	M	60	HBV/ HDV	HCC	2	18, 17	Grade II	Grade II	Yes	A	11	381	3.8	2.3
9	F	81	HCV	HCC	1	81	No	Grade I	No	B	9	8.6	3.7	3.0
10	M	47	HC	HCC	1	57	No	No	No	B	5	5.6	0.6	4.3

*HBV* hepatitis B virus, *Eth* ethanol,, *HDV* hepatitis D virus, *HCV* hepatitis C virus, *HC* hemochromatosis, *HCC* hepatocellular carcinoma, *CAC* cholangiocarcinoma; *Mets*, metastases, *EV* esophageal varices, *Asc* ascites, *Enc* encephalopathy, *BCLC* Barcelona clinic liver cancer staging, *C-P* Child–Pugh score, *AFP* alpha-fetoprotein (ng/mL), *Bili* total bilirubin (mg/dL), *Alb* albumin (g/dL), *UK* unknown, *Dysp Nod* dysplastic nodules.

**Table 2:** Cirrhosis patient details.

Patient	Sex	Age (year)	Etiology	CT/MRI	Tumor number	EV	Asc	Enc	C-P	AFP	Bili	Alb
11	M	57	Eth	No	0	Grade I	Grade I	No	6	4.2	1.8	4.4
12	M	63	HBV	No	0	Grade I with bleeding	Grade I	Yes	9	9.8	4.0	3.7
13	M	46	HCV	No	0	No	No	No	5	6.4	1.1	5.3
14	M	69	UK	No	0	Grade I	Grade I	No	6	?	1.1	3.6
15	F	60	Eth	No	0	No	No	No	5	?	1.2	3.9
16	M	73	Eth	No	0	No	No	No	5	2.4	2.2	4.0
17	M	50	HCV	Dysp Nod	2	Grade I	No	Yes	6	42.6	0.8	4.4
18	M	59	Eth + HBV	Dysp Nod	Multiple	Grade II with bleeding	Grade III	Yes	15	1.5	5.3	2.0
19	M	50	HCV	No	0	Grade I	No	Yes	6	?	1.4	4.5
20	F	59	Eth + HCV	No	0	No	No	No	5	2.7	1.1	5.3
21	M	56	HBV	Yes	0	Grade I	No	Yes	7	4.5	2.3	4.1
22	M	46	HCV	No	0	No	No	No	5	?	0.8	4.8
23	M	77	HCV	Yes	0	Grade I	No	Yes	6	?	1.0	4.2
24	F	53	HCV	No	0	Grade I	No	Yes	6	23.1	1.5	3.8
25	M	80	Eth	No	0	No	No	No	5	?	0.6	4.1

For abbreviations, see Table 1.

**Table 3:** Comparison of clinical variables for liver cirrhosis (LC) and hepatocellular carcinoma (HCC) patients.

Variable	LC	HCC	P value
Males/females	12/3	7/3	0.57
Age (years)	59.9 ± 10.7	65.4 ± 11.6	0.19
Albumin	4.14 ± 0.79	3.45 ± 0.75	0.04
AFP	10.8 ± 13.6	89.7 ± 159	0.11*
Total bilirubin	1.75 ± 1.30	2.06 ± 1.27	0.55
Thrombocytes	131,867 ± 54,014	122,900 ± 41,983	0.66
Glucose	113 ± 45	103 ± 21	0.49
AST	51.9 ± 34.9	104 ± 63	0.003*
ALT	50.3 ± 58.9	99.3 ± 89.5	0.003*
INR	1.32 ± 0.54	1.26 ± 0.20	0.74

For continuous variables, mean ± standard deviation is given with comparisons made using unpaired t tests.

For categorical variables, comparisons were made using the chi-squared test.

*Analyzed by nonparametric Mann Whitney U test due to non-Gaussian distribution of data.

**Figure 3:**
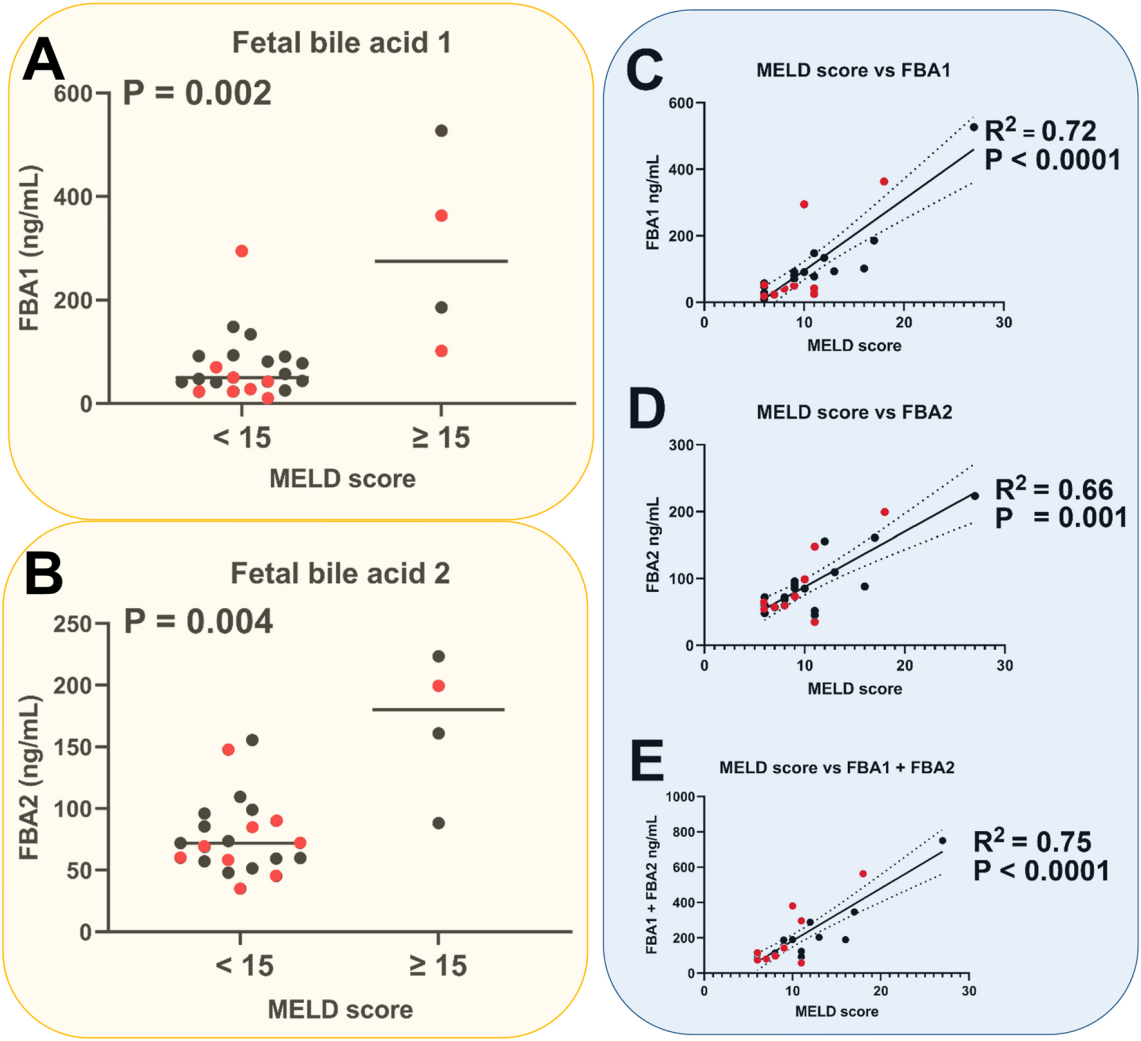
Statistically significant relationship (nonparametric Kruskal–Wallis test) between MELD score and (**A**) fetal bile acid **1** (FBA1) plasma concentration (ng/mL), (**B**) fetal bile acid **2** (FBA2) plasma concentration (ng/mL) and statistically significant correlations (simple linear regression) between MELD score and (**C**) FBA1 plasma concentration, (**D**) FBA2 plasma concentration and (**E**) the sum of FBA1 and FBA2 plasma concentrations. Black symbols represent LC patients without HCC and red symbols represent LC patients with HCC. Dotted lines represent 95% confidence intervals.

As can be seen from these findings, plasma concentrations of both fetal bile acids correlated increased significantly with severity of cirrhosis as measured by Child–Pugh class (Fig. 2A,B) and correlated highly statistically significantly (P < 0.0001) with severity of cirrhosis as determined by the Child–Pugh score (Fig. 2C,D). Moreover, the total fetal bile acid plasma concentration (FBA1 + FBA2) correlated highly statistically significantly (P < 0.0001) with Child–Pugh score (Fig. 2E). When nonparametric correlation was used for the data in Fig. 2C–E, Spearman rank correlation coefficients of 0.87, 0.74 and 0.87, respectively, were found, which were highly statistically significant (P < 0.0001). In contrast to the Child–Pugh score, the MELD score (Model for End-stage Liver Disease) ranges from 6 to 40 and is a validated predictor of survival in end-stage liver disease, such as decompensated cirrhosis. Plasma concentrations of **1** and **2** were statistically significantly higher in patients with MELD scores ≥ 15 than those with values < 15^17^ (Fig. 3A,B). The value of 15 has been reported as a cutoff for predicting short-term survival. That notwithstanding, plasma concentrations of FBA1, FBA2 and FBA1 + FBA2 all correlated statistically significantly with MELD score (Fig. 3C–E). When nonparametric correlation was used for data in Fig. 3C–E, Spearman rank correlation coefficients of 0.76, 0.61 and 0.73, respectively, were found, which were highly statistically significant (P < 0.0001 to 0.001).

In addition, the 3-oxo-Δ^4^ steroids shown in Fig. 1 are both precursors of CDCA that are metabolized by AKR1D1, 7α-hydroxy-cholest-4-en-3-one early in the neutral pathway and 7α-hydroxy-3-oxochol-4-en-24-oic acid (**2**) late in the acidic pathway^13^. Statistical analysis of product/substrate ratios in patient plasma, specifically, CDCA/FBA1, CDCA/FBA2 and CDCA/(FBA1 + FBA2) revealed no statistically significant correlations with Child–Pugh scores (data not shown). Importantly, the presence of HCC did not affect the plasma concentrations of the fetal bile acids, which appeared to be related to severity of cirrhosis as determined by the Child–Pugh criteria. For FBA1, median plasma concentrations with and without HCC were 53.8 and 83.1 ng/mL, respectively (P = 0.50). For FBA2, these values were 72.1 and 85.6 ng/mL, respectively (P = 0.64). Moreover, plasma concentrations of alpha-fetoprotein (AFP; Tables 1,2), a protein made by fetal liver and used as a diagnostic aid for HCC^18^, did not correlate with plasma FBA1 and FBA2 concentrations, either alone or combined (data not shown). AFP values, as expected were statistically significantly higher for patients with HCC (P = 0.007).

**Figure 4:**
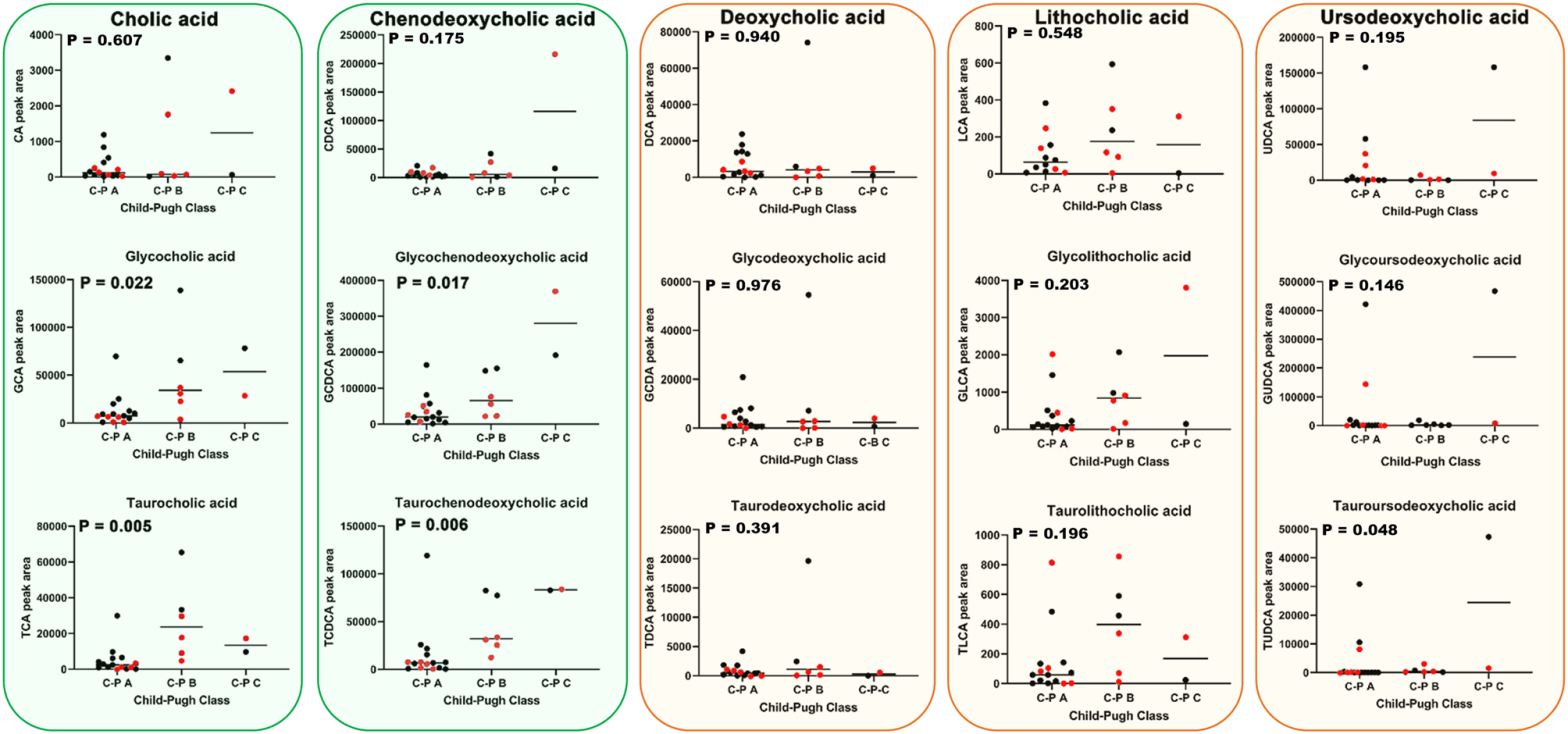
Relationships (Kruskal–Wallis test) between plasma area responses of 15 bile acids and severity of cirrhosis determined by Child–Pugh classes A, B and C. Green boxes represent the primary bile acids cholic acid and chenodeoxycholic acid. Brown boxes represent the secondary bile acids deoxycholic acid, lithocholic acid and ursodeoxycholic acid. Each free bile acid is grouped together with its glycine and taurine conjugates. C-P mean Child–Pugh. Black symbols represent LC patients without HCC and red symbols represent LC patients with HCC.

In addition to the determination of plasma fetal bile acids, 15 primary and secondary bile acids (free and glycine and taurine conjugated) were determined in patient plasma by UPLC-QTOFMS. Figure 4 depicts area responses for each of these plasma bile acids in relation to the Child–Pugh classes A, B and C.

Moreover, correlations for the 15 free and conjugated primary and secondary bile acids were also sought using the Spearman rank correlation due to the non-Gaussian distribution of the bile acid and MELD score data. After Bonferroni correction for multiple comparisons, only the taurine conjugated primary bile acids TCA (Spearman r = 0.669; adjusted P = 0.008) and TCDCA (Spearman r = 0.602; adjusted P = 0.03) correlated statistically significantly. These were stronger correlations than those with the Child–Pugh score). The other 13 bile acids did not correlate with the MELD score with Spearman r values from 0.029 to 0.469 (adjusted P values from 0.08 to 1.0). In addition, an analysis of the 15 bile acids was undertaken in relation to the presence or absence of each of ascites, encephalopathy and esophageal varices. None of these 45 analyses conducted using the nonparametric Mann–Whitney U test produced statistically significantly differences after Bonferroni correction for multiple comparisons.

As for the fetal bile acids in Figs. 2 and 3, the presence of HCC did not influence the relationship between plasma bile acids and Child–Pugh class. Although the conjugated primary bile acids appeared to increase with increasing cirrhosis severity, when P-values were adjusted for multiple comparisons using the Bonferroni correction, no statistically significant relationships remained for these primary bile acid conjugates. In addition, when these 15 primary and secondary bile acids were compared between liver cirrhosis patients with and without HCC, there were no statistically significant differences found (Table 4).

**Table 4:** Lack of effect of the presence of HCC on 15 primary and secondary bile acids in patients with liver cirrhosis (LC).

Bile acid	LC with HCC	LC without HCC	P value
**Free and conjugated primary bile acids (median values)**			
Cholic acid	80	215	0.56
Glycocholic acid	8350	12,698	0.28
Taurocholic acid	4743	6071	0.78
Chenodeoxycholic acid	5208	5564	0.69
Glycochenodeoxycholic acid	27,001	33,628	0.61
Taurochenodeoxycholic acid	9771	15,457	0.74
**Free and conjugated secondary bile acids (median values)**			
Deoxycholic acid	3003	5874	0.19
Glycodeoxycholic acid	1444	4787	0.12
Taurodeoxycholic acid	276	863	0.12
Lithocholic acid	116	75	0.88
Glycolithocholic acid	334	140	0.69
Taurolithocholic acid	119	59	0.52
Ursodeoxycholic acid	3928	1249	0.83
Glycoursodeoxycholic acid	1575	1395	0.74
Tauroursodeoxycholic acid	210	77	0.88

Analyzed by nonparametric Mann–Whitney U test due to non-Gaussian distribution of data.

In summary, plasma concentrations of fetal bile acids 1 and 2 alone and combined were highly correlated with cirrhosis severity (Figs. 2, 3). This was not so for the 15 primary and secondary bile acids also determined in patient plasma (Fig. 4). It is therefore probable that the metabolic activity of AKR1D1 declines with increasing severity of cirrhosis in a manner that is unaffected by the presence of hepatic tumors.

## Discussion

As their name suggests, fetal bile acids occur predominantly in the fetus and are known to decline significantly in the first few days after birth^9^. However, elevated concentrations of these 3-oxo-Δ^4^ bile acids have been detected in neonates and children with congenital Δ^4^-3-oxosteroid 5β-reductase (AKR1D1) deficiency^10, 11, 19, 20^. Tentative identification of fetal bile acids **1** and **2** in adults was made in a plasma metabolomics and lipid profiling investigation of HCC patients that used age-matched AML cases as controls. This identification was made on the basis of mass spectroscopic characteristics without authentic chemical standards for **1** and **2**^7^. Apart from this report of 20 HCC patients *vs*. 22 acute myeloid leukemia (AML) controls^7^, these fetal bile acids had only previously been reported in neonates^11, 20^.

Because these authentic standards are not commercially available, a detailed account of their chemical synthesis is provided here, together with characterization of the intermediates by ^1^H NMR and of the two final products **1** and **2** by ^1^H NMR and high-resolution mass spectrometry. Determination of **1** and **2** in children and neonates with Δ^4^-3-oxosteroid 5β-reductase (AKR1D1) deficiency^10, 11, 19, 20^ has typically been performed using urinary analysis by negative ion fast atom bombardment mass spectrometry (FAB-MS) and gas chromatography-mass spectrometry (GC–MS) in a complex assay that involved hydrolysis of bile acid conjugates in urine after overnight incubation with bile salt hydrolase and double derivatization by methylation and trimethylsilylation^10, 11^. We describe here for the first time a rapid and simple quantitative UPLC-TQMS assay for the fetal bile acids **1** and **2**, which has been utilized to investigate plasma from patients with various degrees of liver cirrhosis, both with and without HCC. This investigation established several unique conclusions. First, plasma concentration of the two fetal bile acids was not related to the presence of hepatic tumors. This could be due to small patient numbers, requiring a larger study to solve this issue. In a previous metabolomic study^7^, plasma levels of **1** and **2** were upregulated relative to AML but it was unclear if this was due to HCC or LC. Second, plasma concentrations of the fetal bile acids correlated strongly with increasing both Child–Pugh and MELD scores, indicating a relationship between these bile acids and severity of cirrhosis. Third, a further 15 both primary and secondary bile acids and their glycine and taurine conjugates were neither correlated with Child–Pugh classes A, B and C nor with the MELD score, once allowance for multiple comparisons was made. These findings were also unrelated to the presence of HCC. These results point to plasma fetal bile acids as the only indication of severity of cirrhosis known so far among the bile acid family.

The highlights of this study were that we describe a step-by-step chemical synthesis of these two fetal bile acids, which are not commercially available. In addition, we have described a simple, rapid and quantitative liquid chromatography-tandem mass spectrometry assay for their quantitation in human plasma. We have applied these novel methodologies to the investigation of hepatic cirrhosis. The limitations of our study concern this last component. First, we recruited relatively few patients (n = 25) with HCC (n = 10) and without HCC (n = 15). This group of patients was highly heterogeneous in relation to their complications arising from decompensation, with encephalopathy (n = 7), ascites (n = 8) and esophageal varices (n = 9). Notwithstanding this heterogeneity, clear correlations between each fetal bile acid and both the Child–Pugh score and the MELD score were obtained. A further limitation of this investigation is that liver tissue was not available from the patients and therefore it was not possible to determine *AKR1D1* expression by qPCR or AKR1D1 protein by western blotting. This would have permitted us to confirm the role of AKR1D1 in fetal bile acid disposition, which we posited is compromised with increasing severity of cirrhosis.

Because of the large volume of research on fetal bile acids and inborn Δ^4^-3-oxosteroid 5β-reductase (AKR1D1) deficiency, it is tempting to propose that deteriorating liver function may also affect the activity of this hepatic enzyme leading to elevated fetal bile acids as a phenocopy of the inborn error of metabolism. It has been postulated^10^ that excessive amounts of Δ^4^-3-oxo bile acids might also be expected to occur in children with severe liver diseases such as hepatitis B and autoimmune chronic active hepatitis but not in more benign chronic cholestatic syndromes such as biliary atresia. In this report, a single patient aged 15 months with a deficiency in fumarylacetoacetase (FAH), known as hereditary tyrosinemia type I that is associated with liver cirrhosis and fatal HCC at a young age^21^, had the highest ratio of 7α,12α-dihydroxy-3-oxo-4-cholenoic acid (a fetal bile acid) to cholic acid in a series of 25 children and neonates with various liver diseases^10^. These authors suggested that an altered redox state in hepatocytes or an unusual sensitivity of Δ^4^-3-oxosteroid 5β-reductase to liver damage might explain their findings. Elevated plasma bile acids, in particular, both free and conjugated CDCA, have been reported to be elevated in cirrhotic patients relative to healthy volunteers^22^. Total bile acids and total bile acids/cholesterol ratio were found to be elevated in 451 cirrhotic patients compared with 216 non-cirrhotic patients with non-cholestatic chronic HBV infection in China^23^. An investigation of 32 cirrhotic patients and 27 healthy volunteers examined 12 serum bile acids using UPLC-TQMS and reported that serum concentrations of the conjugated primary bile acids, taurocholic acid (TCA), glycocholic acid (GCA) and taurochenodeoxycholic acid (TCDCA) were positively associated with Child–Pugh classification^24^. Our own data would appear to corroborate these observations, with uncorrected P-values for nonparametric analyses of variance (Kruskal–Wallis) of the relationships between plasma TCA, GCA and TCDCA with Child–Pugh class. However, when corrected for false discovery, these relationships ceased to remain statistically significant, as did any relationship between non-fetal bile acids and the MELD score.

In a study of alcoholic cirrhosis in heavy alcohol drinkers, striking increases in taurine conjugated bile acids in serum were observed (50- to 130-fold) with more moderate increases in glycine conjugated bile acids (10- to 20-fold). Increased levels of glycochenodeoxycholic acid (GCDCA), TCDCA, GCA, and TCA were positively correlated with disease progression from Child–Pugh A to C^25^. These dramatic findings were of a much higher order than those in our study. It would be of great interest to know how fetal bile acids concentrations were modified in such patients.

Plasma or serum AFP concentrations are generally regarded as a useful addition to surveillance by ultrasound, CT or MRI imaging for the detection of early HCC and this has been established by a meta-analysis of 32 studies involving 13,367 patients^26^. Perusal of the raw data establishes that the AFP values for our patients are not diagnostic for the presence of HCC. AFP was detected in 9/15 cirrhotic patients without HCC with a median value of 2.6 ng/mL compared with 9.0 ng/mL for those with HCC. AFP is well known to vary in HCC from normal values (< 10 ng/mL) to extremely high values (> 100,000 ng/mL). When used diagnostically, an AFP of 20 ng/mL shows good sensitivity but low specificity and in contrast a value of 200 ng/mL has a high specificity but a sensitivity of only 22%^27^. It is interesting to propose that deteriorating liver function in the direction of HCC represents an evolving fetal phenotype, particularly when considering the fetal bile acid changes reported here. However, there was a lack of correlation between AFP and fetal bile acid concentrations. In addition, the inborn error of metabolism involving Δ^4^-3-oxosteroid 5β-reductase (AKR1D1) deficiency that leads to the accumulation and urinary excretion of fetal bile acids **1** and **2**^11, 19^, has not been investigated in relation to increased postnatal serum AFP concentrations. The role of an evolving fetal phenotype in LC that may lead to HCC is an open question.

The issue remains how does severity of liver cirrhosis influence the plasma concentration of fetal bile acids **1** and **2**? A generalized increased bile acid synthesis, as eluded to above, may result in elevated plasma concentrations of the intermediates **1** and **2**. Alternatively, as is the case with the inborn error of metabolism with genetically impaired Δ^4^-3-oxosteroid 5β-reductase deficiency, AKR1D1 activity may be impaired by an as yet undefined mechanism, leading to worsening cirrhosis. In human liver biopsies taken from 34 obese patients, *AKR1D1* mRNA expression was reported to decrease with advancing steatosis, fibrosis and inflammation^14^. Glucocorticoids, including endogenous cortisol, both downregulate AKR1D1 and are metabolized by it^28^. A reduced plasma clearance of cortisol in cirrhosis has long been observed^29^, congruent with a slower metabolic turnover of cortisol in cirrhotic patients^30^. Taken together, these and other reports may provide an biochemical basis for the association of increased fetal bile acids in patients with advancing cirrhosis.

## Materials and methods

### Reagents

All reagents for the chemical syntheses that were conducted in South Korea were purchased from Sigma-Aldrich Korea Ltd. and Tokyo Chemical Industry Co., Ltd.

### General methods for chemical purification and analysis

Product purification was conducted by preparative flash column chromatography using ZEOprep 60 unbonded silica (230–400 mesh). All NMR experiments were carried out using a Bruker Avance III 400 MHz NMR spectrometer equipped with a 5-mm broadband-observed probe head. Chemical shifts were measured relative to residual solvent peaks as an internal standard set to δ 7.26 (CDCl_3_). Multiplicities in the ^1^H NMR spectra as described as s = singlet, d = doublet, t = triplet, q = quartet, m = multiplet, br. s = broad singlet. Liquid chromatography-mass spectra (LCMS) were measured in positive electrospray ionization (ESI) mode on a Waters Xevo G2-XS instrument.

### Synthesis of fetal bile acids

**Figure 5:**
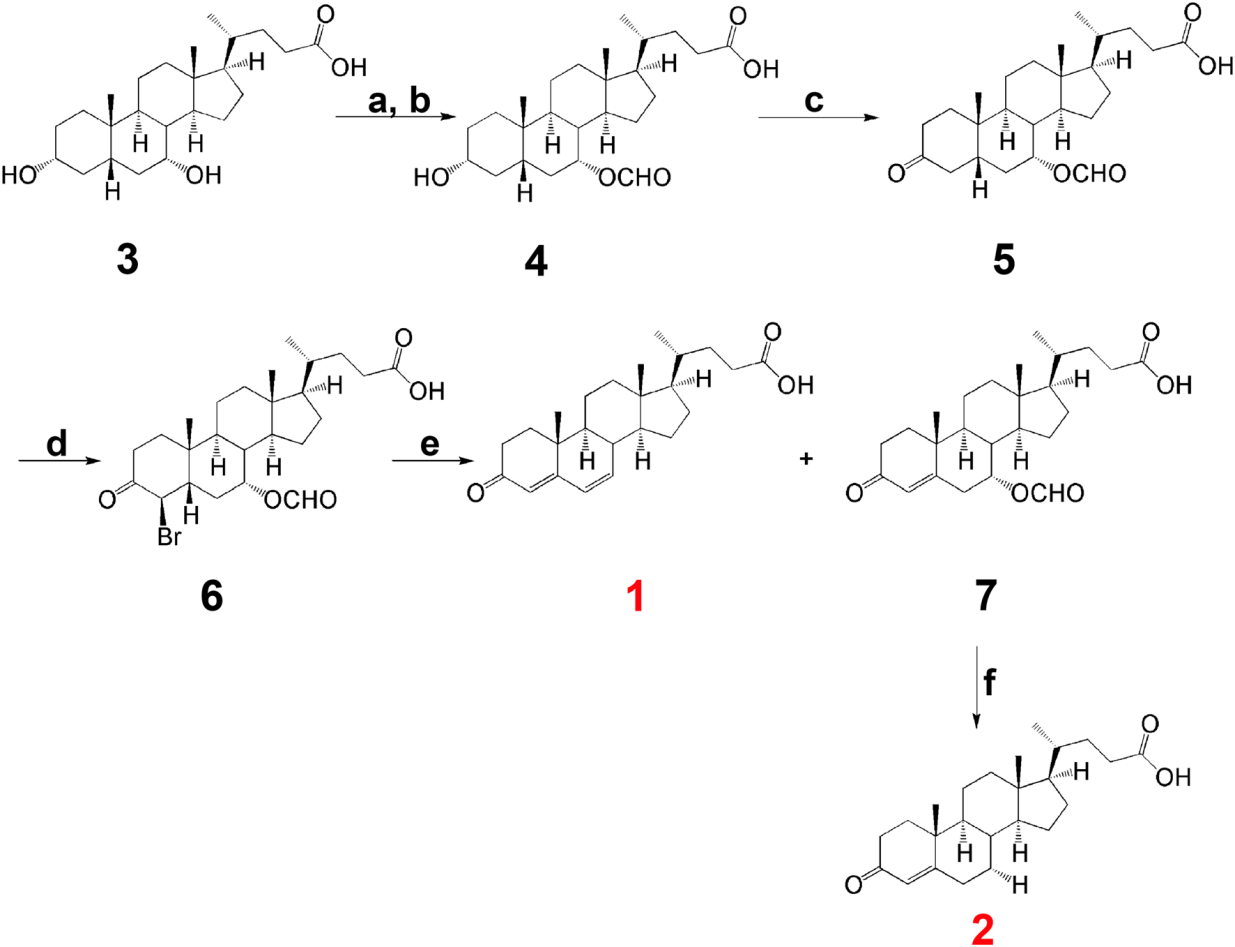
Chemical synthesis of fetal bile acids. Reagents and conditions: a. HCOOH, 70% HClO_4_, 40–60 °C, 2 h; b. 0.2 M NaOH, acetone, RT, 1 h; c. Jones’ reagent, acetone, 0 °C, 2 h; d. PTSA, Br_2_, DMF, RT, 12 h; e. semicarbazide, NaOAc in H_2_O, AcOH, 60 °C, 1 h; f. 0.2 M NaOH, H_2_O, RT, 0.25 h. The reactions were modified from the procedures of Leppik for cholic and deoxycholic acids^31^.

Figure 5 shows the scheme for the synthesis of fetal bile acids **1** and **2**.

Procedures a and b for the synthesis of **4** from **3** (chenodeoxycholic acid) were conducted as follows: a. Compound **3** (2.80 g, 7.13 mmol), formic acid (11 mL) and 70% perchloric acid (0.4 mL, 4.93 mmol) were mixed and the reaction stirred at 55 °C for 1.5 h. To the reaction mixture was slowly added acetic anhydride (9 mL) at 40 °C and then stirred at 55 °C until bubbling ceased. The mixture was then cooled to RT and slowly added to water and extracted with ethyl acetate. The separated organic phase was washed with brine, dried over anhydrous MgSO_4_, filtered and concentrated under reduced pressure. The resulting residue was purified using flash column chromatography (SiO_2_, 5% methanol in dichloromethane) to afford the 3,7-diformyl intermediate (0.57 g, 48%). b. To the 3,7-diformyl intermediate (0.22 g, 0.49 mmol) in acetone (4 mL) was slowly added 0.2 M sodium hydroxide (5 mL, 1.0 mmol) and the reaction mixture stirred at RT for I h, neutralized to pH 7 with acetic acid, diluted with water and extracted with dichloromethane. The separated organic phase was washed with brine, dried over anhydrous MgSO_4_, filtered and concentrated under reduced pressure. The resulting residue was purified using flash column chromatography (SiO_2_, 30% ethyl acetate in n-hexane) to afford compound **4** (0.11 g, 53%).

Procedure c for compound **5** was conducted as follows: To compound **4** (0.11 g; 0.26 mmol) in acetone (11 mL) was slowly added Jones’ reagent (0.3 mL) at 4 °C and the reaction stirred at this temperature for 2 h, then at RT for 1 h, whereupon methanol (0.6 mL) was slowly added to the reaction mixture to form a suspension, which was filtered and washed with acetone. The combined filtrate was concentrated under reduced pressure and the resulting residue purified using flash column chromatography (SiO_2_, 30% ethyl acetate in n-hexane) to afford compound **5** (0.05 g, 46%).

Procedure d for compound **6** was conducted as follows: To compound **5** (0.55 g, 1.31 mmol) and *p*-toluenesulfonic acid (PTSA; 0.04 g, 0.22 mmol) in DMF (5 mL) was slowly added bromine (0.24 g, 1.50 mmol) in DMF (1 mL) at RT and stirred for 20 h. The mixture was then slowly added to water and extracted with ethyl acetate. The separated organic phase was washed with brine, dried over anhydrous MgSO_4_, filtered and concentrated under reduced pressure. The resulting residue was purified using flash column chromatography (SiO_2_, 40% ethyl acetate in n-hexane) to afford compound **6** (0.57 g, 87%).

Procedure e for compounds **1** and **7** was conducted as follows: A solution of semicarbazide.HCl (0.25 g, 2.24 mmol) and sodium acetate (0.18 g, 2.19 mmol) in water (1 mL) was added dropwise to compound **6** (0.41 g, 0.82 mmol) in acetic acid (8 mL) and the mixture stirred at 60 °C for 0.5 h, then stirred at RT for 1 h to form a precipitate. This solid was filtered, washed with pure water and used without further purification. This solid, acetic acid (9 mL), pyruvic acid (0.9 mL) and water (2.1 mL) were stirred at RT for 20 h. Precipitated solid was filtered off and the filtrate extracted with ethyl acetate, which was washed with brine and dried over anhydrous MgSO_4_, filtered and evaporated under reduced pressure. The resulting residue was purified using flash column chromatography (SiO_2_, 50% ethyl acetate in n-hexane) to afford compound **1** (0.025 g, 8%) and compound **7** (0.085 g, 26%).

Procedure f for compound **2** was conducted as follows: Compound **7** (0.085 g, 0.21 mmol) was dissolved in 0.5 M sodium hydroxide (10 mL) and stirred at RT for 1.5 h. The reaction mixture was acidified to pH 1–2 with 1 M HCl and extracted with ethyl acetate. The combined organic phase was washed with brine, dried over anhydrous MgSO_4_ and evaporated under reduced pressure. The resulting residue was lyophilized without further purification to afford compound **2** (0.075 g, 90%).

Spectral data for synthetic fetal bile acids **1** and **2** were as follows:

- (R)-4-((8S,9S,10R,13R,14S,17R)-10,13-dimethyl-3-oxo-2,3,8,9,10,11,12,13,14,15,16,17-dodecahydro-1H-cyclopenta[a]phenanthren-17-yl)pentanoic acid (**1**). m.p. 198–200 °C. ^1^H NMR (400 MHz, CD_3_OD) δ 6.15 (dd, *J* = 9.7, 2.0 Hz, 1H), 6.07 (dd, *J* = 9.8, 2.7 Hz, 1H), 5.55 (s, 1H), 2.60–2.47 (m, 1H), 2.32–2.08 (m, 4H), 2.03–1.92 (m, 3H), 1.90–1.82 (m, 1H), 1.80–1.68 (m, 2H), 1.66–1.57 (m, 1H), 1.54–1.44 (m, 1H), 1.43–1.28 (m, 4H), 1.26–1.10 (m, 5H), 1.06 (s, 2H), 1.00 (d, *J* = 16.3 Hz, 1H), 0.91–0.83 (m, 3H), 0.71 (s, 1H), 0.68–0.61 (m, 1H).; LCMS (ESI): Exact mass calculated for C_24_H_34_O_3_ [M + H]^+^ 371.2586, found 371.2603 (Δm/z = 4.6 ppm).
- (4R)-4-((8S,9S,10R,13R,14S,17R)-7-hydroxy-10,13-dimethyl-3-oxo-2,3,6,7,8,9,10,11,12,13,14,15,16,17-tetradecahydro-1H-cyclopenta[a]phenanthren-17-yl)pentanoic acid (**2**). m.p. 228–230 °C (literature^32^ 227–229 °C). ^1^H NMR (400 MHz, CD_3_OD) δ 5.64 (s, 1H), 3.83 (d, *J* = 2.5 Hz, 1H), 2.62–2.53 (m, 1H), 2.46–2.05 (m, 5H), 2.02–1.91 (m, 2H), 1.91–1.78 (m, 1H), 1.75–1.61 (m, 3H), 1.59–1.18 (m, 9H), 1.16–1.03 (m, 6H), 0.87 (d, *J* = 6.5 Hz, 3H), 0.67 (s, 2H); LCMS (ESI): Exact mass calculated for C_24_H_36_O_4_ [M + H]^+^ 389.2692, found 389.2706 (Δm/z = 3.6 ppm).

Spectral data for the intermediates 4, 5, 6 and 7 are given in the Supplementary Files.

### Quantitation of fetal bile acids 1 and 2 in plasma by ultraperformance liquid chromatography-triple quadrupole mass spectrometry (UPLC-TQMS)

Human plasma (25 μL) was protein precipitated with methanol (475 μL) and centrifuged for 3 min at 25,000 g. Resultant supernatant (100 μL) was diluted with deionized water (100 μL) and 10μL injected into the UPLC-TQMS system, which comprised an Acquity I-Class UPLC and a Xevo-TQ-S Micro mass spectrometer (Waters). UPLC separation was achieved on a CORTECS T3 2.7 μm (2.1 × 30 mm) analytical column with a flow rate of 1.3 mL/min. Mobile phases A and B were 0.01% aqueous formic acid and isopropanol/acetonitrile (50:50 v/v) containing 0.01% formic acid, respectively. After an initial 0.1 min at 30% B, the bile acids were eluted with a gradient of 30–65% B over 0.7 min, followed by a 0.9 min column wash with 98% B and re-equilibrated to initial conditions. The analytical column was maintained at 60 °C. Multiple Reaction Monitoring (MRM) analyses were performed in negative electrospray ionization mode. Ion source temperature and capillary voltage were held constant at 150 °C and 2.0 kV, respectively. The cone gas flow was 50 L/h and desolvation temperature was 650 °C. Fetal bile acids **1** and **2** eluted at 1.08 and 0.82 min, respectively. For fetal bile acid **1**, the MRM used was 369.3 > 325.4 with a dwell time of 0.069 s, a cone voltage of 100 V and a collision energy of 23 eV. For fetal bile acid **2**, the MRM used was 387.3 > 369.4 with a dwell time of 0.069 s, a cone voltage of 100 V and a collision energy of 19 eV. The internal standard (IS) employed was 3β,7α-dihydroxy-5-cholesten-26-oic acid (DHCA) added at a concentration of 500 ng/mL, which eluted at 0.43 min. The MRM for the IS used was 401 > 331 with a cone voltage of 60 V and a collision energy of 20 eV. Peak area ratios to the internal standard were used for construction of calibration curves. For fetal bile acid 1 (FBA1), the calibration curve was linear (R^2^ = 0.998; P < 0.0001). For fetal bile acid 2 (FBA2), the calibration curve was also linear (R^2^ = 0.999; P < 0.0001). Precursor and product ions were similar to a published method but with lower collision energies^33^. The advantage of this assay is the short retention times (< 1.2 min) for **1** and **2**, compared with 33.2 and 29.0 min, respectively, as reported earlier^33^.

### Quantitation of primary and secondary bile acids in plasma by ultraperformance liquid chromatography-triple quadrupole mass spectrometry (UPLC-TQMS)

Human plasma (100 μL) was protein precipitated with methanol (400 μL) and centrifuged at 25,000×*g* for 3 min. Resultant supernatant (100 μL) was diluted with deionized water (100 μL) and 10 μL injected into the UPLC-TQMS system (see above). UPLC separation was achieved on a CORTECS T3 2.7 μm (2.1 × 30 mm) analytical column with a flow rate of 1.3 mL/min. Mobile phases A and B were 0.01% aqueous formic acid containing 0.2 mM ammonium formate and isopropanol/acetonitrile (50:50 v/v) containing 0.01% formic acid and 0.2 mM ammonium formate, respectively. After 0.1 min at 20% B, the bile acids were eluted with a gradient of 20–55% B over 0.7 min, followed by a 0.9 min column wash with 98% B and re-equilibrated to initial conditions. The analytical column was maintained at 60 °C. MRM analyses were performed as above. Retention times together with MRM transitions, cone voltages and collision energies are shown in Supplementary Table S1. Comparison of patients for these 15 bile acids was made from the peak area responses in plasma without calculation of formal concentration using calibration curves^34^. However, in the case of CDCA, the primary bile acid most closely related to FBA1 and FBA2, a calibration curve was constructed as for the fetal bile acids above. For CDCA, the calibration curve was linear (R^2^ = 0.998; P < 0.0001).

### Patient investigations

Patients were recruited from the Octavian Fodor Institute for Gastroenterology and Hepatology Hospital, Cluj-Napoca, Romania. All patients gave their written informed consent prior to study inclusion. The study was performed in accordance with the 1975 Declaration of Helsinki and approved by the institutional review board of the Regional Institute of Gastroenterology and Hepatology “Prof. Dr. Octavian Fodor”, Cluj-Napoca, Romania (approval no. 2445/12.02.2016). All patients included in this study were hospitalized, having been admitted for liver cancer screening, because of a first decompensation event (ascites, encephalopathy, jaundice or variceal bleeding), because of newly diagnosed focal liver lesions on a background of liver cirrhosis or because of referral for treatment (e.g. ablation, surgery or TACE). HCC patients (n = 10) were consecutively enrolled into the study. During the same period, consecutive patients with liver cirrhosis (LC) but no HCC (n = 15) were included. The diagnosis of HCC was made according to non-invasive criteria or based on histology in patients with atypical lesions on a background of liver cirrhosis. LC patients included those on the first diagnosis of cirrhosis based on ultrasound, laboratory data, Fibroscan, CT/MRI and/or hepatic venous pressure measurement with liver biopsy or patients who were known with a history of liver cirrhosis and presented with cirrhosis decompensation (hepatic encephalopathy, ascites, variceal bleeding). The exclusion criteria were: (1) previous or concurrent cancer; (2) active infections other than chronic hepatitis B or hepatitis C virus; (3) history of organ allograft; (4) pregnancy or breastfeeding; and (5) refusal to sign for informed consent. Clinical details of HCC patients and LC patients are given in Tables 1 and 2 respectively. For the HCC patients, 3/10 were female and for the LC patients, 3/15 were female. Child–Pugh class^16^ for the HCC patients (number/total) were A (5/10), B (4/10) and C (1/10) and for the LC patients were A (12/15), B (2/15) and C (1/15), corresponding to Child–Pugh scores^16^ of 5–11 for HCC and 5–15 for LC. MELD scores for the HCC patients ranged from 6 to 18 and for the LC patients ranged from 6 to 27. Peripheral venous blood was drawn into heparinized tubes for each patient and spun at 1300 g to prepare plasma, which was aliquoted and frozen at − 80 °C prior to shipment to Switzerland by courier on dry-ice. Plasmas were stored at − 80 °C in Bern before forwarding of aliquots by courier on dry-ice to Wilmslow, UK where they were analyzed by UPLC-TQMS for fetal bile acids **1** and **2**, together with 15 free and conjugated primary and secondary bile acids as described above.

### Data analysis

Statistical analysis was performed using GraphPad Prism 9.1.0 (San Diego, CA, USA) by nonparametric statistics because of the general non-Gaussian distribution of the data. Comparisons between two groups of data were analyzed using the Mann–Whitney U test and between three or more groups using the Kruskal–Wallis test. Correlations between variables were made by Spearman’s rank correlation. All values are expressed as medians. P < 0.05 was considered statistically significant. The Bonferroni correction was used when multiple comparisons were made.

## Supplementary Information

Supplementary Information.

## Data Availability

Requests for raw data or authentic compounds should be made to the corresponding author (jeff.idle@liu.edu).
