# Supplementary material for: Plasma fetal bile acids 7α-hydroxy-3-oxochol-4-en-24-oic acid and 3-oxachola-4,6-dien-24-oic acid indicate severity of liver cirrhosis
Source: Sci Rep. 2021 Apr 15;11:8298. doi: 10.1038/s41598-021-87921-5 (PMC8050265; doi:10.1038/s41598-021-87921-5)
Supplement: Supplementary file 1 — Supplementary Information. [file 41598_2021_87921_MOESM1_ESM.pdf]

Plasma fetal bile acids 7α-hydroxy-3-oxochol-4-en-24-oic acid and 3-oxachola-4,6-dien-24-oic acid indicate the severity of liver cirrhosis

Tudor Mocan, Dong Wook Kang, Billy J. Molloy, Hyeonho Jeon, Zeno A. Spârchez, Diren Beyoğlu and Jeffrey R. Idle

Supplementary Materials

Supplementary spectral data

Compound 4 (Fig. 4)

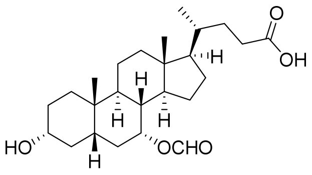

(*R*)-4-((3*R*,5*S*,7*R*,8*R*,9*S*,10*S*,13*R*,14*S*,17*R*)-7-(formyloxy)-3-hydroxy-10,13-dimethylhexadecahydro-1*H*-cyclopenta[*a*]phenanthren-17-yl)pentanoic acid (4).
Prepared according to the procedure b. ^{1}H NMR (400 MHz, $CDCl_3$) δ 8.08 (d, *J*=1.0 Hz, 1H), 5.02 (d, *J*=3.2 Hz, 1H), 3.50 (td, *J*=11.0, 5.5 Hz, 1H), 2.40 (ddd, *J*=15.4, 10.1, 5.2 Hz, 1H), 2.26 (ddd, *J*=15.9, 9.6, 6.6 Hz, 1H), 2.17 (s, 2H), 2.07–1.94 (m, 3H), 1.83 (tdd, *J*=13.7, 7.4, 3.6 Hz, 3H), 1.72–1.40 (under water peak, m, 7H), 1.40–1.23 (m, 6H), 1.22–0.97 (m, 4H), 0.96–0.91 (m, 4H), 0.90–0.78 (m, 1H), 0.66 (s, 2H).

Compound 5 (Fig. 4)

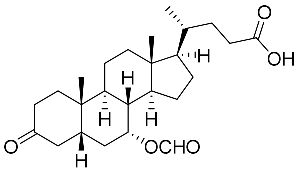

(*R*)-4-((5*R*,7*R*,8*R*,9*S*,10*S*,13*R*,14*S*,17*R*)-7-(formyloxy)-10,13-dimethyl-3-oxohexadecahydro-1*H*-cyclopenta[*a*]phenanthren-17-yl)pentanoic acid (5).
Prepared according to the procedure c. ^{1}H NMR (400 MHz, $CDCl_3$) δ 8.06 (d, *J*=1.0 Hz, 1H), 5.12 (d, *J*=3.2 Hz, 1H), 3.02 (dd, *J*=15.2, 13.8 Hz, 1H), 2.46–2.32 (m, 2H), 2.32–2.24 (m, 1H), 2.24–2.16 (m, 1H), 2.15–2.06 (m, 2H), 2.04–1.92 (m, 3H), 1.91–1.77 (m, 3H), 1.73–1.65 (m, 3H), 1.60–1.52 (m, 2H), 1.51–1.30 (m, 7H), 1.29–1.23 (m, 1H), 1.22–1.07 (m, 2H), 1.04 (s, 2H), 0.95 (d, *J*=6.5 Hz, 2H), 0.70 (s, 2H).

Compound 6 (Fig. 4)

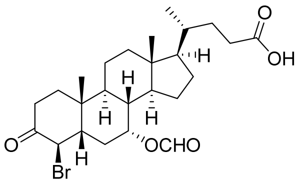

(*R*)-4-((4*R*,5*S*,7*R*,8*S*,9*S*,10*R*,13*R*,14*S*,17*R*)-4-bromo-7-(formyloxy)-10,13-dimethyl-3-oxohexadecahydro-1*H*-cyclopenta[*a*]phenanthren-17-yl)pentanoic acid (6). Prepared according to the procedure d. ^{1}H NMR (400 MHz, $CDCl_3$) δ 8.08 (d, *J*=4.7 Hz, 1H), 5.34 (d, *J*=11.8 Hz, 1H), 5.17 (d, *J*=3.2 Hz, 1H), 2.65–2.49 (m, 2H), 2.41 (ddd, *J*=15.4, 10.0, 5.2 Hz, 1H), 2.27 (ddd, *J*=17.8, 9.6, 6.6 Hz, 1H), 2.21–2.13 (m, 2H), 2.09–2.01 (m, 1H), 2.01–1.93 (m, 2H), 1.93–1.70 (m, 3H), 1.61–1.50 (m, 2H), 1.50–1.31 (m, 5H), 1.30–1.22 (m, 1H), 1.22–1.12 (m, 2H), 1.11 (s, 2H), 0.98-0.91 (m, 3H), 0.70 (s, 2H).

Compound 7 (Fig. 4)

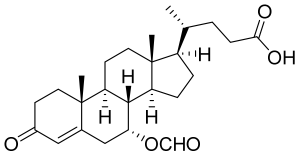

(*R*)-4-((7*R*,8*S*,9*S*,10*R*,13*R*,14*S*,17*R*)-7-(formyloxy)-10,13-dimethyl-3-oxo-2,3,6,7,8,9,10,11,12,13,14,15,16,17-tetradecahydro-1*H*-cyclopenta[*a*]phenanthren-17-yl)pentanoic acid (7). Prepared according to the procedure e. ^{1}H NMR (400 MHz, $CDCl_3$) δ 8.05 (d, *J*=3.8 Hz, 1H), 5.17 (s, 1H), 2.68–2.50 (m, 1H), 2.50–2.33 (m, 2H), 2.33–2.19 (m, 2H), 2.20–1.99 (m, 3H), 1.93–1.67 (m, 4H), 1.64–1.42 (m, 5H), 1.39–1.07 (m, 9H), 1.01–0.87 (m, 3H), 0.79–0.65 (m, 2H).

UPLC-TQMS characteristics of 16 free and conjugated bile acids

Supplementary Table S1. Details of the ultraperformance liquid chromatography-triple quadrupole mass spectrometry assay for 15 free and conjugated bile acids

Bile acid	Retention Time (min)	MRM transition	Cone voltage (V)	Collision energy (eV)
Lithocholic acid (LCA)	0.90	375.25 > 375.25	60	32
Deoxycholic acid (DCA)	0.83	391.25 > 391.25	60	16
Glycolithocholic acid (GLCA)	0.82	432.25 > 74.00	60	35
Chenodeoxycholic acid (CDCA)	0.80	391.25 > 391.25	60	16
Taurolithocholic acid (TLCA)	0.71	482.25 > 80.00	60	60
Glycodeoxycholic acid (GDCA)	0.70	448.25 > 74.00	60	35
Glycochenodeoxycholic acid (GCDCA)	0.67	448.25 > 74.00	60	35
Cholic acid (CA)	0.66	407.25 > 343.25	60	34
Ursodeoxycholic acid (UDCA)	0.66	391.25 > 391.25	60	16
Taurodeoxycholic acid (TDCA)	0.60	498.25 > 80.00	60	60
Taurochenodeoxycholic acid (TCDCA)	0.57	498.25 > 80.00	60	60
Glycocholic acid (GCA)	0.55	464.25 > 74.00	60	34
Taurocholic acid (TCA)	0.46	514.25 > 80.00	60	64
Tauroursodeoxycholic acid (TUDCA)	0.44	498.25 > 80.00	60	60
Glycoursodeoxycholic acid (GUDCA)	0.52	448.25 > 74.00	60	35

Primary bile acids (CA and CDCA) and their glycine and taurine conjugates are shaded green.
